# The “Torment” of Surgical Antibiotic Prophylaxis among Surgeons

**DOI:** 10.3390/antibiotics10111357

**Published:** 2021-11-06

**Authors:** Massimo Sartelli, Federico Coccolini, Angeloantonio Carrieri, Francesco M. Labricciosa, Enrico Cicuttin, Fausto Catena

**Affiliations:** 1 Department of Surgery, Macerata Hospital, 62100 Macerata, Italy; angeloantoniocarrier@libero.it; 2 Department of General, Emergency and Trauma Surgery, Pisa University Hospital, 56100 Pisa, Italy; federico.coccolini@gmail.com (F.C.); enrico.cqtn@gmail.com (E.C.); 3 Global Alliance for Infections in Surgery, 62100 Macerata, Italy; labricciosafrancesco@gmail.com; 4 General and Emergency Surgery Department, Bufalini Hospital, 47521 Cesena, Italy; faustocatena@gmail.com

**Keywords:** surgical antibiotic prophylaxis, surgical site infections, antimicrobial resistance, antimicrobial prescription

## Abstract

Surgical antibiotic prophylaxis (SAP) is one of the peri-operative measures for preventing surgical site infections (SSIs). Its goal is to counteract the proliferation of bacteria in the surgical site during intervention in order to reduce the risk of SSIs. SAP should be administered for surgical interventions where the benefit expected (prevention of SSIs) is higher compared to the risk (serious side effects, such as acute kidney injury, *Clostridioides* *difficile* infection, and the spread of antimicrobial resistance). In prescribing SAP, surgeons should have both the awareness necessary “to handle antibiotics with care”, and the knowledge required to use them appropriately.

## 1. Introduction

One of the goals of best practice across surgical pathways is to prevent surgical site infections (SSIs). SSIs are infections that occur up to 30 days after surgical operation or up to one year after surgical intervention in patients receiving prosthetic materials. They can affect either the superficial tissues or the deep tissues at the operation site. Superficial SSIs can affect both the skin and subcutaneous tissues, while deep SSIs can involve tissues under the skin such as muscle, fascia, and organs/space opened during the surgical operation [1].

SSIs are the costliest and second most common healthcare-associated infections (HAIs), being the first among surgical patients. They are associated with a longer post-operative hospital length of stay and result in higher post-operative mortality and morbidity. Most SSIs can be preventable by basic infection prevention and control measures [2,3].

Preventing SSIs should be considered a global priority since in the latest few decades bacteria have become resistant to antibiotics, making the prevention of SSIs very important in the containment of antibiotic resistance.

Despite great progresses in infection prevention and control in the previous years, nowadays SSIs continue to represent one of the most frequent adverse events in surgical departments worldwide, and their prevention requires the integration of basic measures before, during, and after the surgical operation.

Surgical antibiotic prophylaxis (SAP) [4] is one of the most important peri-operative measures for preventing SSIs. The goal of SAP is to achieve serum and tissue drug levels for the duration of the surgical operation that exceed the minimum inhibitory concentration in order to counteract the proliferation of microorganisms likely to be encountered during the intervention [5]. Ideally, the antibiotic should be administered as close to the incision time as possible to reduce the risk of SSIs. [6].

SAP should be administered for surgical interventions where the benefit expected (prevention of SSIs and their potential severity) is higher compared to the risk (serious side effects, such as acute kidney injury, *Clostridioides difficile* infection, and spread of antimicrobial resistance).

SAP is the most common indication for antibiotic use in hospitals around the world [7], and approximately 15% of all antibiotics used in hospitals are prescribed for SAP.

Although SAP is one of the most important measures to prevent SSIs, antibiotics alone are not sufficient to prevent SSIs. SAP does not replace good surgical techniques, infection prevention and control measures, or peri-operative optimization of the patients’ risk factors [8,9,10].

Many surgeons believe that SAP is peripheral to their role. In prescribing antibiotics, surgeons should have the awareness “to handle antibiotics with care”, and the knowledge to use them appropriately [11,12,13]. Surgeons prescribing SAP have two potentially conflicting responsibilities. First, surgeons should offer optimal antibiotic coverage for the individual patient. Second, they both should preserve the efficacy of antibiotics and minimize their adverse effects.

Clinical practice guidelines for SAP have been published by the American Society of Health-System Pharmacists (ASHP), the Infectious Diseases Society of America (IDSA), the Surgical Infection Society (SIS), and the Society for Healthcare Epidemiology of America (SHEA) [14]. However, high rates of SAP prescribing practices not compliant with guidelines are common, and may contribute to sub-optimal patients’ outcomes, cause adverse effects, and be an important driver of antimicrobial resistance [15].

The guidelines recommend that SAP should be administered to prevent infections before and only during surgical intervention. Antibiotics should not be administered after surgical operation, as often happens. Probably the most common mistake made by surgeons in prescribing SAP is to prolong its administration in the post-operative period. However, it is well known that long duration of SAP (more than 24 h) does not result in an additional decrease in SSIs rate in any procedures but, on the contrary, are associated with increasing adverse effects, such as the risk of acute kidney injury and the risk of *Clostridioides difficile* infection [16]. Moreover, incorrect timing of SAP reduces its efficacy [2], while its inappropriate use, in terms of prolonged duration, can lead to select for resistant microorganisms and to high costs [6,15].

For many surgeons, SAP is not a priority. The poor awareness of surgeons about SAP is well demonstrated in the literature. Broom et al. [17], in a study involving twenty surgeons and anesthetists from a tertiary referral hospital in Australia, investigated experiences and perspectives on SAP prescribing, suggesting the need for intra-specialty (within surgeons) and inter-specialty (among surgeons, anesthetists, and infectious diseases specialists) intervention strategies to address the barriers to the implementation of guidelines. An Italian study demonstrated that only 18.1% of the patients received appropriate antibiotic prophylaxis based on choice, dose, and duration of antibiotics [18]. Antibiotic prescription was perceived as peripheral to their role by surgeons in a British study [19], while other studies highlighted lack of motivation and time to develop non-surgical skills [20].

Ideally, an appropriate SAP should have six main characteristics: (1) preventing SSIs, (2) improving the patients’ outcomes, (3) reducing the duration and related cost of health care, (4) reducing adverse effects, such as acute kidney injury or the risk of *Clostridioides difficile* infection, (5) reducing consequences for the patient’s microbial flora, and (6) reducing the emergence of antibiotic resistance.

The selected antibiotic for SAP should be: (1) active against the bacteria that most likely can contaminate the surgical site, (2) administered in an appropriate dosage and at the correct time, in order to obtain appropriate tissue concentrations during the period of potential contamination during the surgical intervention, (3) safe, and (4) administered for the shortest period to minimize the adverse effects, the emergence of antibiotic resistance, and costs and, at the same time, to maximize its efficacy.

In the next section of our paper, we briefly outline the principles that a surgeon should follow in prescribing SAP correctly.

## 2. Principles of Appropriate SAP

SAP should be administered for all of those operative procedures having a high risk of post-operative SSIs, or when prosthetic materials are implanted. The surgical interventions can be divided into four classes according to the degree of bacterial contamination and the consequent incidence of post-operative infections [18]. For elective surgical interventions, SAP is recommended in selected cases of clean surgery and in all cases of clean-contaminated surgery. In the case of contaminated surgery, the choice to perform prophylaxis rather than therapy will be evaluated separately for each type of surgical intervention or situation based on the available evidence [21]. For dirty surgery, starting antibiotic therapy immediately is recommended [22].

The implantation of any prosthetic material increases the risk of infection of the surgical site, as it reduces the host’s defenses [23]. In fact, in the presence of prosthetic material, a low bacterial load may be sufficient to cause infection. Usually, SAP is recommended when the surgical operation involves the implantation of prosthetic material in clean surgery.

SAP should be effective against the bacteria that most likely contaminate the surgical site. Contamination of the surgical site is a frequent occurrence during surgery. In many cases it is the inevitable consequence of a surgical technique. In other cases, it is the consequence of a violation of asepsis techniques. Contamination can be both endogenous and exogenous.

Endogenous contamination occurs when the responsible microorganisms are the commensals present on the skin such as *Staphylococcus aureus* and *Staphylococcus epidermidis* or normal flora colonizing the incised mucosae such as *Escherichia coli*, or another *Enterobacterales* or anaerobes. Exogenous contamination is caused by microorganisms that meet the patient accidentally, generally coming from the surgical team, the operating room environment, and the instruments, and cannot be predicted a priori.

The antibiotic chosen for prophylaxis should have a spectrum of action that guarantees efficacy against the most probable contaminants. It has been shown that the effectiveness of prophylaxis is limited to endogenous contaminants; only these pathogens can, in fact, be reasonably predicted and therefore “covered” by antibiotic prophylaxis.

It is crucial to choose antibiotics with the narrowest spectrum of activity required, since broad spectrum antibiotics may be required later if the patients develop serious post-operative infections and may induce the spread of antibiotic resistance.

Intravenous “first generation” cephalosporins such as cephazolin are the most common antibiotics used in SAP. Intravenous ‘second generation’ cephalosporins, such as cefoxitin improving coverage against anaerobic and aerobic Gram-negative antibiotics, can be used as alternative to the combination of “first generation” cephalosporin and metronidazole in elective abdominal surgery.

Routine use of vancomycin in SAP is not generally recommended. Vancomycin should be always considered for SAP in patients with known methicillin-resistant *Staphylococcus aureus* (MRSA) colonization or at high risk for MRSA colonization, such as patients with recent hospitalization or patients coming from nursing-home residents. However, even if vancomycin is commonly recommended when the risk for MRSA is high, it is important to point out that the evidence suggests that vancomycin is less effective than first generation’ cephalosporins for preventing SSIs caused by methicillin-susceptible *Staphylococcus aureus* [14].

Intravenous administration of the antibiotic within 120 min is generally recommended. Administration of the first dose of antibiotic 30 to 60 min before surgical incision is usually suggested for most used antibiotics (e.g., cefazolin) in order to ensure appropriate tissue concentrations during the operative period. A single dose is generally sufficient. Additional intra-operative doses should be administered for procedures exceeding two half-lives of the antibiotic or with associated significant blood loss (more than 1.5 L).

It is generally accepted as good clinical practice that the dose of the antibiotic used for prophylaxis is the same as that used for therapy. This dose must guarantee appropriate tissue concentrations at the time of the surgical incision [24].

Intravenous administration of the antibiotic within 120 min prior to incision is generally recommended. However, for the most used antibiotics, such as cefazolin, intravenous administration 30–60 min prior to incision is the most reliable method to ensure an effective concentration of the antibiotic in the tissues at the site of the intervention [25].

Many of the antibiotics used in SAP have relatively short half-lives (1–2 h) calculated in healthy volunteer studies. It is therefore logical to administer an additional dose of antibiotic if the surgical procedure lasts more than 2–4 h to ensure adequate serum and tissue concentrations of the antibiotic. Additional intra-operative doses are generally recommended if the duration of the operative procedure exceeds two half-lives of the antibiotic or there is excessive blood loss (more than 1.5 L) [8].

The shortest effective duration of SAP for preventing SSI is not known. However, evidence have shown that post-operative antibiotic administration is not for most procedures and should be less than 24 h for most procedures.

Erroneously, the surgeon believes that prolonging antibiotic “coverage” can protect the patient from infections that can develop after surgery and protect from possible medico-legal repercussions in case of infectious complications. However, the surgeon often ignores that all of the studies that have demonstrated the efficacy of antibiotic prophylaxis were aimed exclusively at preventing peri-operative infections (and SSIs), not infections that occur in the post-operative course and nosocomial infections that develop away from the surgical site.

The shortest effective duration of antibiotic prophylaxis for preventing SSI is not known. Evidence has shown that post-operative antibiotic administration is not for most procedures and should be less than 24 h for most procedures [14].

## 3. Conclusions

In conclusion, SSIs continue to pose an important clinical challenge in surgical departments worldwide. It is essential to recognize that much of the burden of prolonged hospitalizations, increased healthcare costs, mortality, and morbidity associated with SSIs is preventable through the integration of basic measures before, during, and after the surgical operation. SAP is one of the most important peri-operative measures for preventing SSIs; the principles of SAP are clearly established, and several guidelines for them have been published. However, high rates of inappropriate SAP, in terms of timing and duration, has been demonstrated across the surgical pathway. Adherence to evidence for SAP should be improved by multidisciplinary intervention strategies, both intra-specialties, actively involving surgeons, and inter-specialty, directly connecting surgeons, anesthetists, infectious diseases specialists, epidemiologists, and pharmacologists.

One way to engage surgeons in guideline development and implementation is to translate recommendations into a protocol or pathway that specifies and coordinates responsibilities and timing for actions among the multi-disciplinary team. Moreover, since the choice of SAP and its spectrum also depends on local epidemiology, a local adaptation, of evidence-based guidelines for SAP should be developed and implemented in each acute health care facility around the world.

Finally, surveillance of HAIs including SSIs is pivotal for infection prevention and control because it can provide quality data that can be used in monitoring and alert system reducing the incidence of preventable HAIs [26,27,28,29,30]. Facility-based SSI surveillance should be always performed to guide interventions with timely feedback of results to surgeons. The collection and analysis of monitoring data should serve to identify vulnerabilities in the system, this being the basis for infection prevention, control improvement, and risk reduction.
